# Preparation, characterization and application in cobalt ion adsorption using nanoparticle films of hybrid copper–nickel hexacyanoferrate†

**DOI:** 10.1039/c9ra00596j

**Published:** 2019-03-06

**Authors:** Xinxin Long, Rongzhi Chen, Shengjiong Yang, Jixiang Wang, Tijun Huang, Qin Lei, Jihua Tan

**Affiliations:** College of Resources and Environment, University of Chinese Academy of Sciences Yuquan Road 19A Beijing 100049 China crz0718@ucas.ac.cn tanjh@ucas.ac.cn; Key Laboratory of Environmental Engineering, Xi'an University of Architecture and Technology No. 13, Yanta Road Xi'an Shaanxi 710055 China

## Abstract

Different mole ratios (*n*_Cu_ : *n*_Ni_ = *x* : *y*) of hybrid copper–nickel metal hexacyanoferrates (Cu_*x*_Ni_*y*_HCFs) were prepared to explore their morphologies, structure, electrochemical properties and the feasibility of electrochemical adsorption of cobalt ions. Cyclic voltammetry (CV), field emission scanning electron microscopy (FE-SEM), Fourier transform infrared spectroscopy (FTIR) and X-ray diffraction (XRD) indicated that the *x* : *y* ratio of Cu_*x*_Ni_*y*_HCF nanoparticles can be easily controlled as designed using a wet chemical coprecipitation method. The crystallite size and formal potential of Cu_*x*_Ni_*y*_HCF films showed an insignificant change when 0 ≤ *x* : *y* < 0.3. Given the shape of the CV curves, this might be due to Cu^2+^ ions being inserted into the NiHCF framework as countercations to maintain the electrical neutrality of the structure. On the other hand, crystallite size depended linearly on the *x* : *y* ratio when *x* : *y* > 0.3. This is because Cu tended to replace Ni sites in the lattice structure at higher molar ratios of *x* : *y*. Cu_*x*_Ni_*y*_HCF films inherited good electrochemical reversibility from the CuHCFs, in view of the cyclic voltammograms; in particular, Cu_1_Ni_2_HCF exhibited long-term cycling stability and high surface coverage. The adsorption of Co^2+^ fitted the Langmuir isotherm model well, and the kinetic data can be well described by a pseudo-second order model, which may imply that Co^2+^ adsorption is controlled by chemical adsorption. The diffusion process was dominated by both intraparticle diffusion and surface diffusion.

## Abstract 2

Cu_*x*_Ni_*y*_HCF films with appropriate Cu/Ni ratios are expected to be prepared as designed for the recovery of Co^2+^ from spent LIBs.

## Graphical abstract

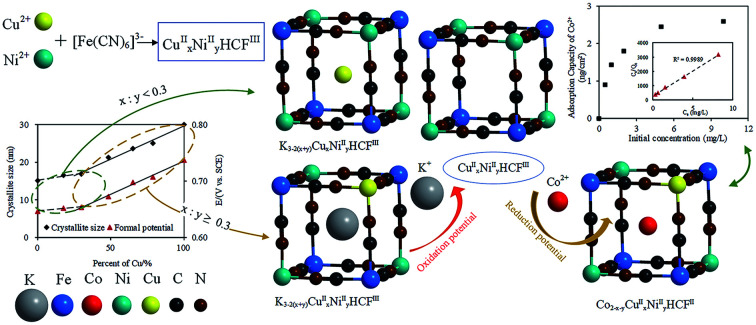

## 1 Introduction

Iron hexacyanoferrate (PB, Prussian blue) and its analogues (PBAs) are collectively referred to as transition metal hexacyanoferrates (MHCFs, M = Fe, Cu, Co, Ni, *etc.*) Due to the electrochromism, magnetism, redox activity, and electrochemical stability associated with their unique cubic structure, MHCFs have been applied in a number of fields, including catalysis,^1^ electrochromics,^2^ electrocatalysis,^3,4^ detection,^5^ chemical sensors,^6^ capacitors,^7^ secondary batteries,^8^ photocatalysis,^9^ photochromics,^10^ biosensors,^11,12^ and metal ion sieving or capture,^13,14^ making great contributions to environmental technology and new-generation energy development.

Recently, MHCFs have received extensive attention due to their unique electrochemical properties as modified electrode materials, such as PB (FeHCF),^15^ copper hexacyanoferrate (CuHCF),^16^ cobalt hexacyanoferrate (CoHCF),^17^ nickel hexacyanoferrate (NiHCF),^18^ and zinc hexacyanoferrate (ZnHCF).^19^ Each MHCF has its own unique electrochemical and spectral properties; however, materials with diversified traits are preferred. Research into hybrid-metal hexacyanoferrates (h-MHCFs) is becoming a trend due to their multiple characteristics and properties. Previous researchers reported that the prepared h-MHCFs existed as a new phase rather than a simple mixture of corresponding single metal hexacyanoferrates.^20,21^ Ghasemi *et al.*^22^ found that NiCoHCF has a higher capacitance than NiHCF or CoHCF; Li *et al.*^23^ revealed that CuNiHCF has a higher capacity retention than CuHCF as a cathode for sodium-ion batteries; it was also reported that the mixed MHCF showed an enhanced catalytic efficiency to H_2_O_2_ as compared to that of FeHCF.^24^ The surface coverage of CoNiHCF was proved to be higher than those of CoHCF and NiHCF.^25^

In an aqueous electrolyte containing alkali salts, MHCFs exhibit reversible redox reactions (such as Fe^III^/Fe^II^) accompanied by reversible intercalation and deintercalation of hydrated alkali cations, low cost, and low toxicity.^26^ Within the family of MHCFs, an NiHCF modified electrode showed an excellent cation exchange capacity in redox reactions due to its open structural framework,^27–29^ with uniform, compact and stable films. The network structures with macropores^26^ of CuHCF films were characterized by nearly ideal current–potential curves.^30^ We also found that a CuNiHCF film modified electrode showed higher electrochemical stability in comparison to CuHCF and NiHCF in phosphate buffer solutions (PBS).^25^ To the best of our knowledge, few papers on different proportions of Cu_*x*_Ni_*y*_HCF nanoparticle films as modified electrode materials have been published, which inspired us to synthesize Cu_*x*_Ni_*y*_HCF films that may inherit the virtues of CuHCF and NiHCF.

Generally, MHCF film modified electrodes are prepared by electrodeposition, and are difficult to produce on a large scale,^25^ and fall off easily. Moreover, it is hard to control the proportion of metal phases (M_1_ : M_2_) in the products (h-MHCFs), which is why few researchers have focused on the characteristic differences among varied proportions of h-MHCFs, such as morphology, structure, or electrochemical properties. Chen *et al.*^31^ prepared exact ratios of CuHCF nanoparticle ink by a wet chemical coprecipitation method, and printed them onto the electrode surface to remove cesium electrochemically; the cesium removal was comparable in magnitude to that by an electrodeposited film. Wang *et al.*^25^ prepared h-MHCF films by the same method; the surface coverage of redox-active sites of the CuCoHCF films was 35 nmol cm^−2^, which was much higher than the 9.02 nmol cm^−2^ of electrodeposited films;^32^ and the Co/Ni atomic ratio in CoNiHCF compounds had been proved to be controlled by the reactant ratio in the reaction mixture.

Herein, we prepared a series of Cu_*x*_Ni_*y*_HCF nanoparticle films with different molar ratios (*n*_Cu_ : *n*_Ni_ = *x* : *y*) by using a wet chemical coprecipitation method, and explored the characteristics of each ratio and the variations among them by multiple characterization methods. Recently, the wide application of lithium-ion batteries (LIBs) has resulted in a rapid increase in the amount of spent LIBs, containing valuable metals such as cobalt, lithium, nickel, and copper. Therefore, we also attempt to recover cobalt ions (Co^2+^) from solutions by using Cu_*x*_Ni_*y*_HCF nanoparticle films. The feasibility of electrochemical adsorption of cobalt ions onto hybrid copper–nickel hexacyanoferrate films is discussed, as well as the adsorption isotherms and kinetics properties.

## 2 Experimental

### 2.1 Materials and apparatus

All reagents, such as Cu(NO_3_)_2_·*x*H_2_O, Ni(NO_3_)_2_·6H_2_O, Co(NO_3_)_2_·6H_2_O, LiNO_3_, K_3_[Fe(CN)_6_], K_3_[Fe(CN)_6_]·3H_2_O and KNO_3_, used in the operations were of analytical grade or better without further purification. Ultrapure water (18 MΩ cm) was used throughout all the operations. All the experiments were carried out at room temperature.

Ion concentrations were determined by inductively coupled plasma mass spectrometry (ICP-MS, Thermo Fisher Scientific iCAP Q, America). The surface morphologies of Cu_*x*_Ni_*y*_HCF films were observed by a field-emission scanning electron microscope (FE-SEM, Hitachi SU-8010, Japan). Their structures and compositions were characterized by X-ray powder diffractometry (XRD, Rigaku SmartLab, Japan) and Fourier transform infrared spectroscopy (FTIR, Bruker Vertex 70, Germany). Cyclic voltammetry of Cu_*x*_Ni_*y*_HCF electrode films was performed by using an electrochemical analyzer (Chen Hua CHI660e, China).

### 2.2 Preparation of modified electrodes

As described in our previous studies,^25^ h-MHCF nanoparticle inks with different metal molar ratios were prepared by wet chemical coprecipitation. Firstly, mixed solutions with different molar ratios (*x* : *y* = 1 : 4, 1 : 2, 1 : 1, 2 : 1, and 4 : 1) were obtained from Cu(NO_3_)_2_·*x*H_2_O and Ni(NO_3_)_2_·6H_2_O, to which a solution of K_3_[Fe(CN)_6_] was added; the reactant mole ratio of total metal nitrates to K_3_[Fe(CN)_6_] was kept at 3 : 2. Secondly, one group of parallel samples were dried in an oven at 60 °C to obtain powder samples for subsequent chemical analysis and characterizations. The remaining samples were surface-treated by K_4_[Fe(CN)_6_]·3H_2_O to dissolve well in water. Thirdly, the 5% (ratio of mass to volume) ink-like solutions of nanoparticles were prepared and shaken for one week (25 °C, 150 rpm). Indium tin oxide (ITO) was washed with acetone as a support substance, and Cu_*x*_Ni_*y*_HCF nanoparticle inks were coated on it using a KW-4A spin-coater instrument (2000 rpm for 10 s followed by 2500 rpm for another 10 s). Finally, the modified electrodes were dried in an oven at 120 °C for 2 h.

### 2.3 Characterization

In order to investigate the composition ratios, the prepared Cu_*x*_Ni_*y*_HCF powders were decomposed in aqua regia by using microwave digestion. After microwave digestion, dilution and filtration, the elements were determined by ICP-MS. The obtained *x* : *y* ratio is linearly fitted to the theoretical values in the synthesis process. The fitness (*R*^2^) is given by eqn (1):1
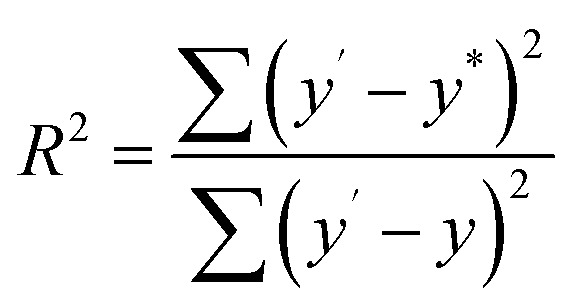
y*, *y* and *y*′ stand for the theoretical ratio values, the actual ratio values and the mean value of *y*, respectively. For convenience, the ratio of *x* : *y* is converted to the percentage of Cu (*W*_Cu_) in the nanoparticle films, with the following expression:2
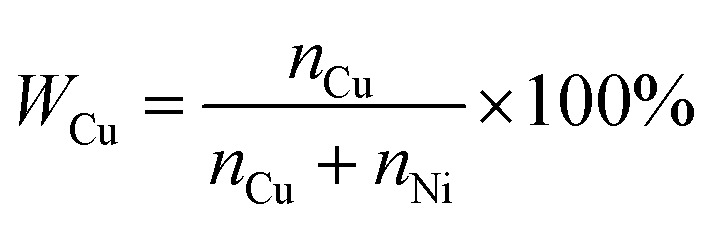
where *n*_Cu_ and *n*_Ni_ stand for the number of moles of Cu and Ni in Cu_*x*_Ni_*y*_HCF nanoparticle films, respectively.

A three-electrode system was used with a saturated calomel electrode (SCE, Hg/Hg_2_Cl_2_/KCl) as the reference electrode, a Pt wire as the counter electrode and the ITO plate loaded with nanoparticle films as the working electrode. The cyclic voltammograms (CVs) of Cu_*x*_Ni_*y*_HCF films in the different cation solutions were obtained, using a CHI660e electrochemical analyzer. The electrochemical properties of Cu_*x*_Ni_*y*_HCF nanoparticle films were characterized by CV, formal potential (*E*_f_), accumulated quantity of electric charge (*Q*) and surface coverage of redox-active sites (*Γ*). *E*_f_ is calculated as half of the sum of potentials of the anodic (*E*_pa_) and cathodic (*E*_pc_) current peak. *Q* is the charge obtained by integrating the cathodic peak after subtraction of the background. The calculation equation of *Γ* is as follows:3
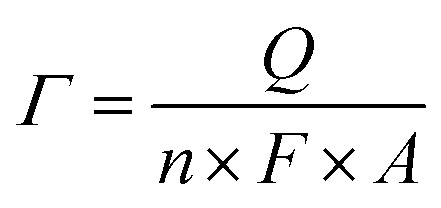
*Q* is obtained by integrating the cathodic peak from the 2nd cycle under the background correction at a scan rate of 0.05 V s^−1^, and other symbols have their usual meanings. The electrodes used here are modified by 3% ink.

The stability of the Cu_*x*_Ni_*y*_HCF films was investigated by long-term potential cycling (500 cycles), expressed by the rate of decrease (*R*_d_) of the integrated Faraday charge *Q* with the increase in the number of cycles.4
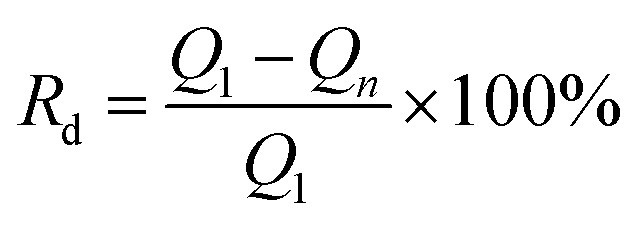
*Q*_1_ and *Q*_*n*_ stand for the integrated charge recorded from the 1st and *n*th scan cycles, respectively.

### 2.4 Electrochemical adsorption of Co^2+^

#### 2.4.1 Removal efficiency

Co^2+^ removal was performed in a three-electrode cell containing 40 mL solutions stirred at 500 rpm by a magnetic stirrer (DL Instruments Ltd., MS-H280-Pro, China). The films were firstly pretreated by an applied potential of +1.3 V (*vs.* SCE) for 30 min in 1 mg L^−1^ KNO_3_ solution to discharge the residual K^+^ during the surface treatment. The relevant reaction is as follows:

5 K_4_[Fe^II^(CN)_6_] → 4K^+^ + [Fe^III^(CN)_6_]^3−^ + e^−^

Afterwards, the films were washed with ultrapure water and transferred to 1 mg L^−1^ Co^2+^ solution to adsorb Co^2+^ by applying a reduction potential of −0.4 V (*vs.* SCE).

6 M_3_[Fe^III^(CN)_6_]_2_ + 2e^−^ + Co^2+^ → CoM_3_[Fe^II^(CN)_6_]_2_

Contrast tests were conducted at the same time without applying potentials.

The results of electrochemical adsorption were given as removal efficiency (*R*), which was calculated according to eqn (7):7
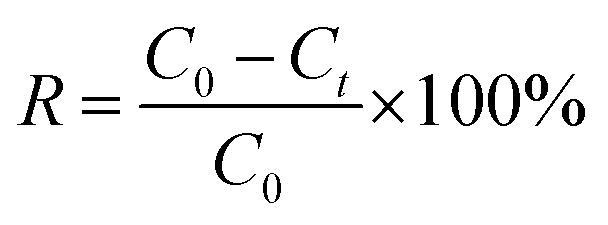
where *C*_0_ (mg L^−1^) and *C*_*t*_ (mg L^−1^) stand for the Co^2+^ concentration at the initial stage and at time *t*, respectively.

#### 2.4.2 Isotherm and kinetic studies

Batch adsorption experiments were conducted in Co^2+^ solutions at various initial concentrations (0.5 mg L^−1^, 1 mg L^−1^, 2 mg L^−1^, 5 mg L^−1^, and 10 mg L^−1^) to investigate the adsorption mechanism. The batch adsorption data were fitted with the Langmuir model (8), and the Freundlich isotherm model (9).8
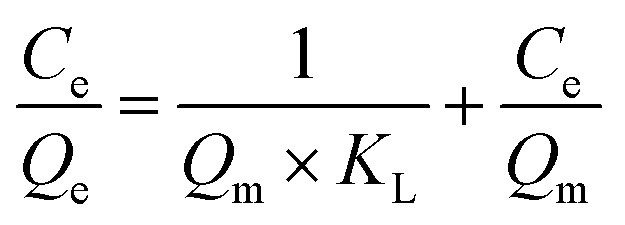
9
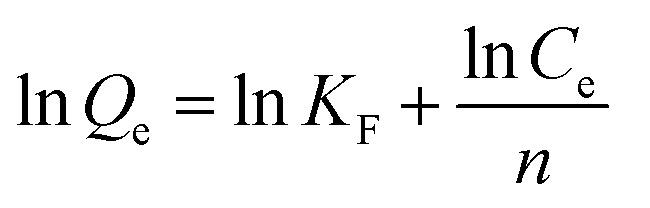


Here, *C*_e_ (mg L^−1^) is the equilibrium concentration; *Q*_e_ (mg cm^−2^) and *Q*_m_ (mg cm^−2^) are the saturation adsorption capacity and the maximum adsorption capacity at the pseudo-equilibrium, respectively. *K*_L_, *K*_F_ and *n* (dimensionless) are the adsorption isotherm constants for the Langmuir isotherm and the Freundlich isotherm.

The intraparticle diffusion model, pseudo-first-order and pseudo-second-order adsorption kinetic models were used to analyze the dynamic adsorption process, as represented by eqn (10)–(12), respectively.

10 *Q*_*t*_ = *k*_p,i_ × *t*^0.5^ + *C*_i_

11 ln(*Q*_e_ − *Q*_t_) = ln *Q*_e_ − *k*_1_ × *t*

12
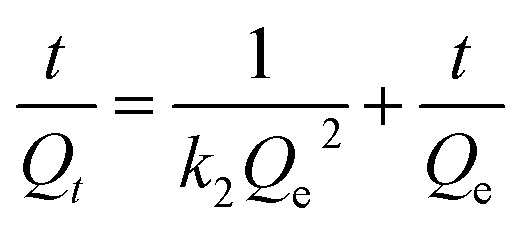
where, *Q*_e_ (mg cm^−2^) and *Q*_*t*_ (mg cm^−2^) are the adsorption amounts of Co^2+^ at the adsorption equilibrium and time *t*, respectively. *k*_p,i_ (i represents the different stages), *k*_1_ and *k*_2_ are the rate constants. *C*_i_ is the intercept at different stages.

## 3 Results and discussion

### 3.1 Characterizations of Cu_*x*_Ni_*y*_HCF nanoparticle films

#### 3.1.1 Component analysis

The actual contents of Cu and Ni of the prepared Cu_*x*_Ni_*y*_HCFs were determined by ICP-MS, as listed in Table 1.

**Table 1:** Atomic number of Cu and Ni in Cu_*x*_Ni_*y*_HCF nanoparticles

*x* : *y*	4 : 1	2 : 1	1 : 1	1 : 2	1 : 4
Cu	0.6282	0.5263	0.3860	0.2562	0.0792
Ni	0.1704	0.2839	0.4130	0.6022	0.3726

The fitness of the theoretical and actual values is 0.9840, showing that the two sets of values are very close. In other words, unlike electrodeposition,^33,34^ the *x* : *y* ratios in the Cu_*x*_Ni_*y*_HCF nanoparticle films can be controlled by the preparation method. This is because hexacyanoferrate (II) ions are generated firstly in the vicinity of the electrode surface and then react primarily with one kind of metal ion in the electrolyte^27^ when prepared by electrodeposition. For coprecipitation, metal ions and hexacyanoferrate (III) ions have been mixed and reacted evenly during the preparation stage of the ink. The results mean that Cu_*x*_Ni_*y*_HCF films with appropriate metal ratios can be synthesized as required.

#### 3.1.2 Morphology

**Fig. 1:**
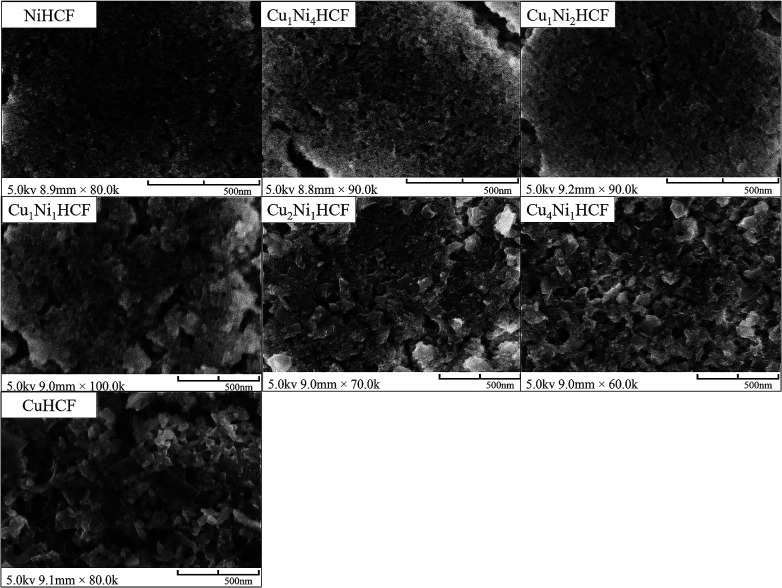
FE-SEM images of Cu_*x*_Ni_*y*_HCF nanoparticle films with different metal ratios (M_1_ : M_2_).

The surface structures of the nanoparticle films are shown in Fig. 1. It is found that NiHCF film exhibits a compact structure consisting of homogeneous nanoparticles, with the formation of evident cracks and the agglomeration of NiHCF nanoparticles. While abundant pores obviously grow on CuHCF films, which might facilitate the mass transfer and diffusion rate of ions within films.^25^ The nanoparticle size of CuHCF films is obviously larger than that of NiHCF films, which is consistent with previous research results.^26,35^ The nanoparticle size and porosity of Cu_*x*_Ni_*y*_HCF films varied from pure NiHCF films to pure CuHCF films. The presence of Ni had a significant effect on the morphology of Cu_*x*_Ni_*y*_HCF films. Cu_4_Ni_1_HCF, Cu_2_Ni_1_HCF, Cu_1_Ni_1_HCF, Cu_1_Ni_2_HCF and Cu_1_Ni_4_HCF displayed particulate agglomeration and stratification, which were similar to that observed in NiHCF films.

#### 3.1.3 Structure

**Fig. 2:**
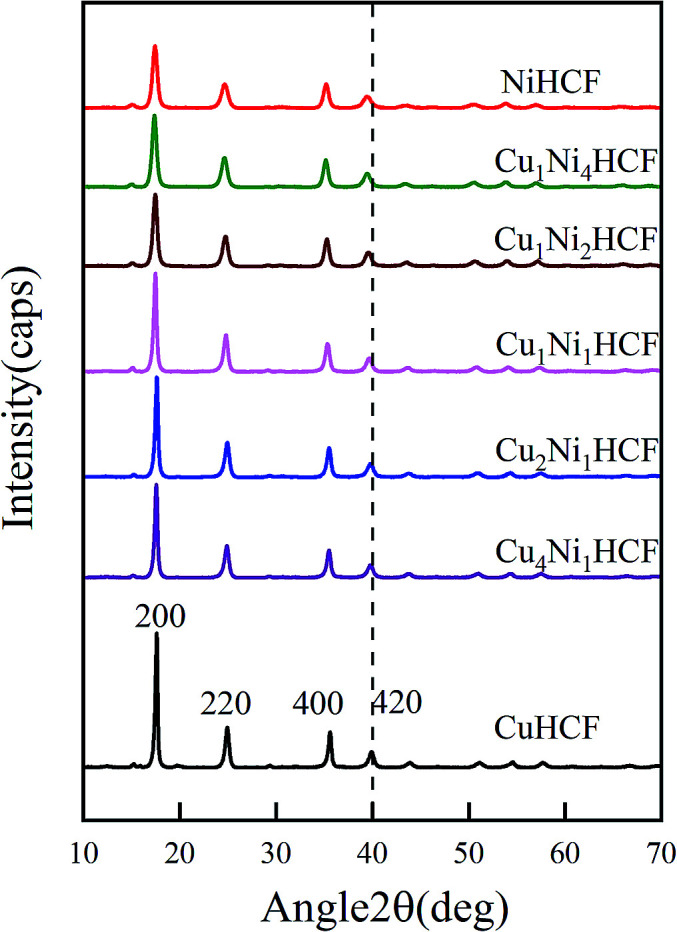
XRD patterns of Cu_*x*_Ni_*y*_HCF powders.

In order to further explore the effect of *x* : *y* ratio on the properties of Cu_*x*_Ni_*y*_HCFs, Cu_*x*_Ni_*y*_HCF powders were characterized by XRD and FTIR. As shown in Fig. 2, all Cu_*x*_Ni_*y*_HCFs have four predominant 2*θ* peaks in the range of 17–40° corresponding to the (2 0 0), (2 2 0), (4 0 0) and (4 2 0) diffraction planes of a face-centered-cubic lattice structure, which are very consistent with the ref. 18, 36 and 37. 2*θ* values obtained for h-MHCFs fall between the NiHCF and CuHCF peak positions. With an increasing content of copper, the diffraction peak position of Cu_*x*_Ni_*y*_HCFs shows a slight shift from NiHCF to CuHCF.

**Fig. 3:**
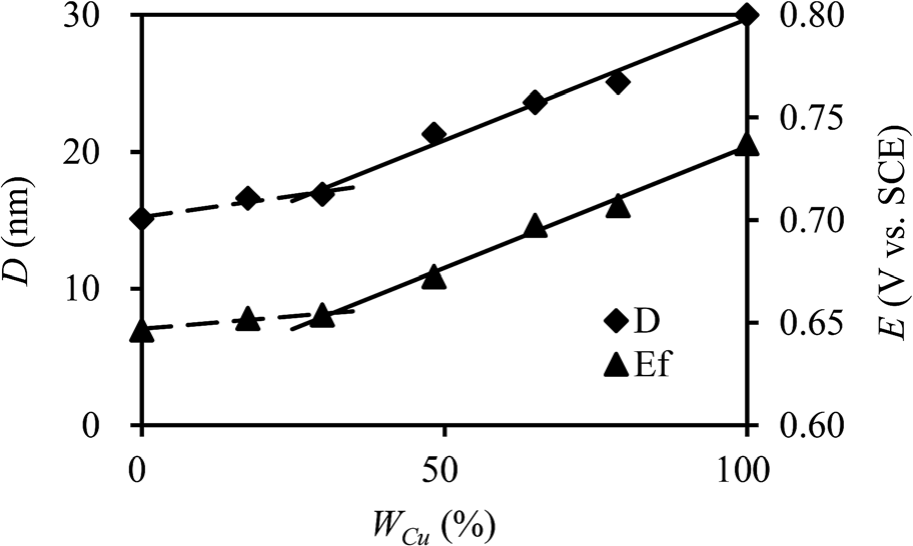
Crystallite size (*D*) and formal potential (*E*_f_) of Cu_*x*_Ni_*y*_HCFs *versus* molar content of Cu (*W*_Cu_).

**Fig. 4:**
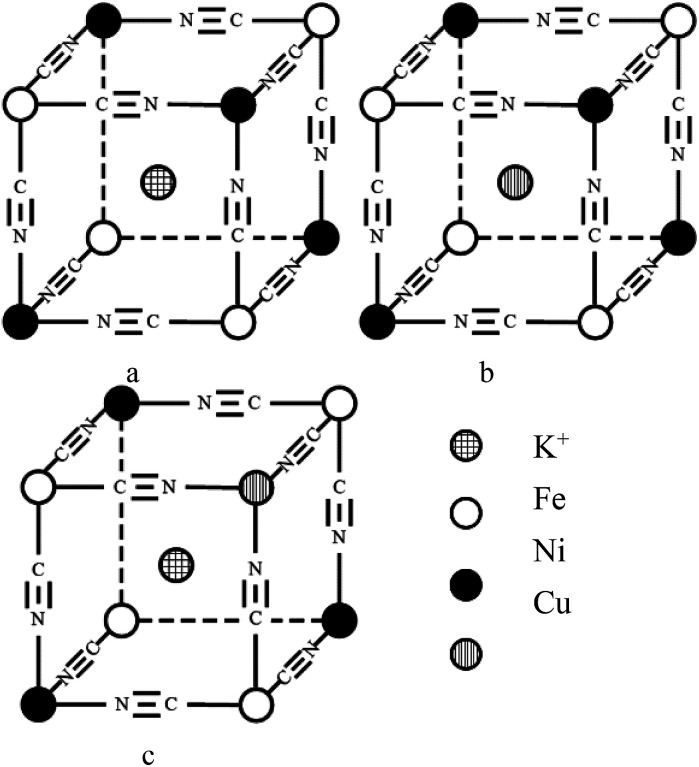
Schematic of 1/8 cell structure of MHCF (M: Cu, Ni).

The crystallite size (*D*) can be deduced from the diffraction peak according to Sherrer's formula:13
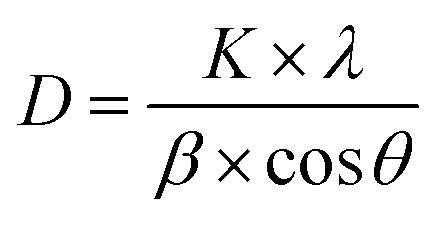
where *K* is the shape factor, *λ* is the X-ray wavelength (0.154056 nm), *β* is the full width at half maximum of the diffraction peak, and *θ* is the Bragg diffraction angle. As presented in Fig. 3, the crystallite sizes of NiHCF and CuHCF are estimated to be about 15 nm and 30 nm, respectively; the sizes of Cu_*x*_Ni_*y*_HCFs at different ratios of Cu/Ni are in between these values. The value of *D* rarely changes with the increase of copper content when *W*_Cu_ < 30%; then it increases linearly with the *W*_Cu_ values. We suppose that the lattice of NiHCF (as shown in Fig. 4a) is practically unchanged when *W*_Cu_ < 33%. Cu^2+^ inset in NiHCF lattice instead of K^+^ as countercations to maintain the electrical neutrality of the structure in a situation such as that exhibited by Fig. 4b. The crystal structure begins to change as the Cu content increases to around 33%, while the Cu replace part of the Ni to occupy the lattice positions illustrated by Fig. 4c.

**Fig. 5:**
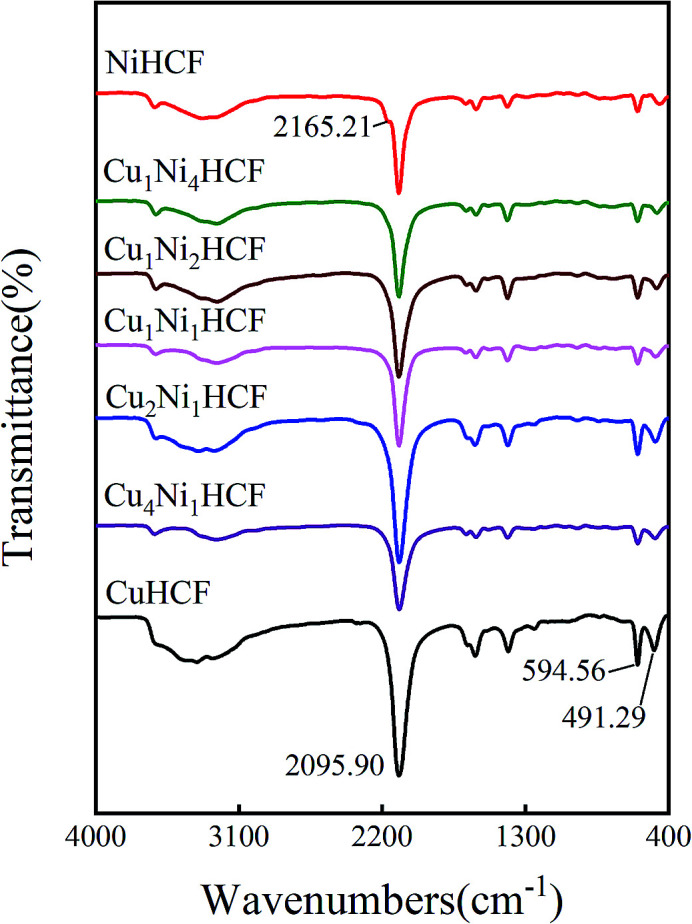
FITR spectrum of Cu_*x*_Ni_*y*_HCF powders.

Fig. 5 illustrates the FTIR spectra of Cu_*x*_Ni_*y*_HCF powders. In particular, there is a small shoulder at 2165.21 cm^−1^ of NiHCF, while the other samples show only one major peak in the vicinity of 2096 cm^−1^. The other bands of Cu_*x*_Ni_*y*_HCFs are very close to each other, owing to the similar absorption peaks of NiHCF and CuHCF. The broad band at around 3330 cm^−1^ refers to the O–H stretching vibrations.^38^ The vibrational band near 2096 cm^−1^ corresponds to the stretching vibration absorption band of the –C≡N group.^39,40^ The bands in the region of 1400–1680 cm^−1^ are related to the asymmetric and symmetric stretching vibrations of COO^−^ groups.^41,42^ The stretching vibration absorption band of the free –C≡N is at about 2060 cm^−1^ in aqueous solution;^43^ the blue shift of the absorption band observed together with the two smaller peaks at about 595 cm^−1^ and 491 cm^−1^ are attributed to the formation of Fe–CN–M.^39,44^

#### 3.1.4 Electrochemical properties

**Fig. 6:**
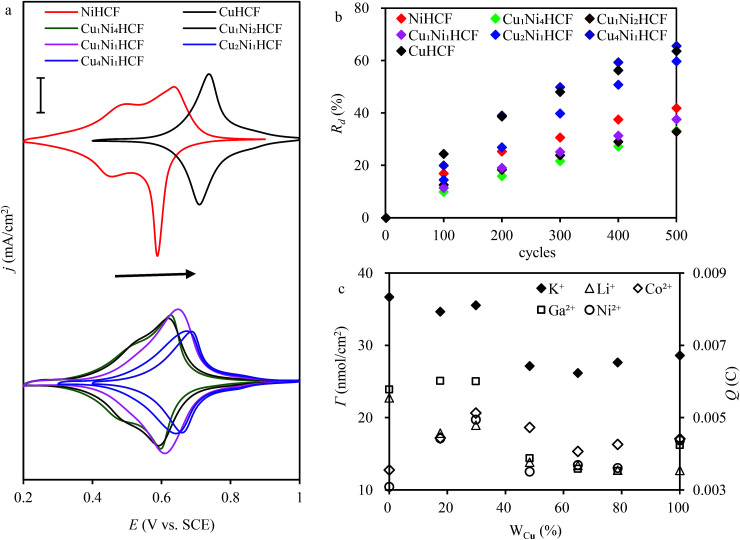
(a) Cyclic voltammograms of Cu_*x*_Ni_*y*_HCF modified electrodes (scan rate: 0.005 V s^−1^; electrode area: 15 mm × 12.5 mm); (b) rate of decrease (*R*_d_) of the cathodic integrated charge *vs.* the number of sweeping cycles. (Scan rate: 0.05 V s^−1^; electrode area: 15 mm × 12.5 mm); (c) surface coverage and integrated Faraday charge in different electrolytes (*c*(K^+^) = 0.5 M, *c*(Li^+^) = 0.5 M, *c*(Co^2+^) = 0.5 M, *c*(Ga^2+^) = 0.1 M, *c*(Ni^2+^) = 0.5 M. Scan rate: 0.05 V s^−1^; electrode area: 15 mm × 12.5 mm).

CV plots with distinct redox couple peaks of Cu_*x*_Ni_*y*_HCF films in 0.5 M K^+^ solution are shown in Fig. 6a. One set of symmetrical redox couples of CuHCF films is around 0.723 V, while the voltammetric patterns of the NiHCF films have two pairs of redox peaks at a lower potential range of 0.45–0.65 V, assigned to the presence of two phases within the NiHCF films.^45,46^ The intensity and location of the response curve peaks of Cu_*x*_Ni_*y*_HCFs depend on the value of *x* : *y*, which is consistent with the change in structure from XRD and FTIR results. As indicated in a previous study,^47^ Cu_*x*_Ni_*y*_HCF is a new phase rather than a simple mixture of CuHCF and NiHCF. The typical CV response of each Cu_*x*_Ni_*y*_HCF is attributed to its respective unique structure. In particular, two sets of redox peaks of Cu_1_Ni_4_HCF and Cu_1_Ni_2_HCF are similar to NiHCF, and, the redox peaks at 0.45 V are severely weakened, which might be because of their special structures, as illustrated in Fig. 4b. The *E*_f_ values of Cu_*x*_Ni_*y*_HCF films were plotted as a function of *W*_Cu_, as approximated in Fig. 3. At a low molar fractions of Cu, the *E*_f_ value hardly changes with the addition of Cu, then depends linearly on the content of copper when *W*_Cu_ rises to about 33%. These observations confirm our previous hypothesis that Cu exists in two forms: as countercations substituted for K^+^, under which condition the *E*_f_ is close to that of NiHCF,^48^ or occupying lattice positions instead of Ni. This is similar to the electrodeposited films studied by Kulesza *et al.*^27^ It can be found from Fig. 6a that the redox peaks at more negative potential disappear when *W*_Cu_ > 30%, which also demonstrates that Cu started to occupy positions of the lattice.

The ratio value of cathodic current to anodic peak (*i*_pc_/*i*_pa_) approaches one, implying the reversibility of the electrochemical redox reaction. The existence of Cu greatly improved the property of NiHCF films in this respect. The long-term CV of Cu_*x*_Ni_*y*_HCF films was carried out in 0.5 M K^+^ at a scan rate of 0.05 V s^−1^ to study the stability of Cu_*x*_Ni_*y*_HCFs. *R*_d_ values were plotted against the cycle numbers, as depicted in Fig. 6b. NiHCF films show comparatively high stability compared with CuHCF films, which is in accord with the materials synthesized previously from chlorides.^25^ The stability of Cu_*x*_Ni_*y*_HCF films increases with a decrease in *x* : *y* values in the order: Cu_4_Ni_1_HCF < Cu_2_Ni_1_HCF < Cu_1_Ni_1_HCF < Cu_1_Ni_2_HCF < Cu_1_Ni_4_HCF. It is noteworthy that the cycling stabilities of Cu_1_Ni_4_HCF, Cu_1_Ni_2_HCF and Cu_1_Ni_1_HCF films are notably better than that of NiHCF films, indicating that the stability of Cu_*x*_Ni_*y*_HCF films will be enhanced on the basis of NiHCF films when the copper content of copper is lower than or even equal to the nickel content. In contrast, the cycling stability will get worse when the proportion of copper is higher.

The results of the surface coverage *Γ* calculated from the integrated charge in 0.5 M K^+^ solution are shown in Fig. 6c. The *Γ* value of NiHCF films is apparently higher than that of CuHCF films. The *Γ* values of Cu_1_Ni_4_HCF films and Cu_1_Ni_2_HCF films are 1.2–1.4 times as much as the *Γ* values of other Cu_*x*_Ni_*y*_HCFs.

### 3.2 Electrochemical adsorption of cations

#### 3.2.1 Electrochemical behaviors in cationic solutions

In addition to K^+^ solution, the CV responses of Cu_*x*_Ni_*y*_HCF films in Li^+^, Co^2+^, Ni^2+^ and Ga^2+^ solutions are also discussed. The electric quantity *Q* of the reduction current of Cu_*x*_Ni_*y*_HCF films in the Li^+^, Co^2+^, Ni^2+^ and Ga^2+^ solutions were integrated (as shown in Fig. 6c). The Cu_1_Ni_2_HCF films show the maximum value of the integrated charge in hybrid films, which may be bound up with the *Γ*.

**Fig. 7:**
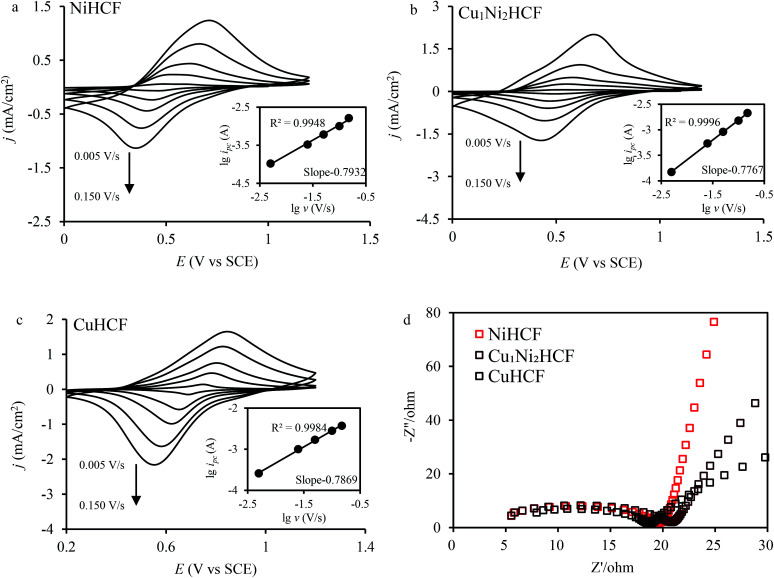
(a, b and c) CV of Cu_*x*_Ni_*y*_HCF films in 0.5 M Co^2+^ solutions at different scan rates (*v* = 0.005, 0.025, 0.05, 0.1, 0.15 V s^−1^); insets show the logarithmic values of reduction peak current (*i*_pc_) as a function of the logarithmic values of scan rate; (d) Nyquist plots of Cu_*x*_Ni_*y*_HCF films from 100 Hz to 1000 kHz.

The fine CV responses imply the possibility of electrochemical adsorption of metal cations. In the electrochemical adsorption experiment, Co^2+^ were effectively removed by Cu_*x*_Ni_*y*_HCFs. That is why Co^2+^ was chosen to carry out the experiment on the electrochemical adsorption behavior of Cu_*x*_Ni_*y*_HCF films. The Cu_1_Ni_2_HCF films were used for comparison with CuHCF films and NiHCF films since they have excellent electrochemical performances. In order to study information about the mechanism, CV and electrochemical impedance spectroscopy (EIS) experiments were conducted. The CVs of Cu_*x*_Ni_*y*_HCF modified electrodes at various sweep rates from 0.005 to 0.15 V s^−1^ in 0.5 M Co^2+^ solutions are shown in Fig. 7a–c. The logarithmic cathodic peak current increases linearly with an increase in the logarithmic scan rate (shown as insets). The slopes of the Cu_*x*_Ni_*y*_HCF films are 0.75–0.88 (listed in Table S1†), indicating that the reaction at the thin films is controlled by an ion diffusion process and a surface electron transfer process, with a tendency towards the latter.^49^ The Δ*E* gradually increases with the variation in the scan rate, indicating a limitation arising from the charge-transfer kinetics^50^ which weakened the reversibility.^51^

The Nyquist plots (Fig. 7d) are composed of a distorted semicircle in the high-frequency region assigned to the charge-transfer reaction of the electrode and a linear part at the low-frequency end due to a diffusion-controlled process.^29,52^ The CuHCF film is characterized by the lowest diameter of the semicircle, standing for the quickest electron transfer rate.^53^ The charge transfer resistance decreases in the order: Cu_1_Ni_2_HCF > NiHCF > CuHCF.

#### 3.2.2 Removal efficiency of Co^2+^

**Fig. 8:**
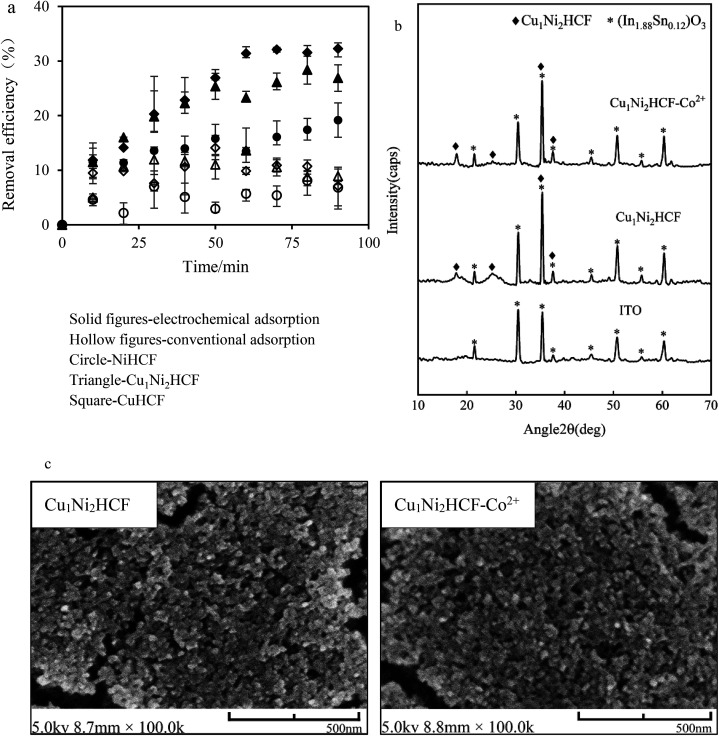
(a) Removal efficiency of Co^2+^ onto Cu_*x*_Ni_*y*_HCF films *vs.* contact time (Co^2+^ concentration: 1 mg L^−1^, electrode area: 15 mm × 25 mm); (b) XRD patterns and (c) SEM images of Cu_1_Ni_2_HCF film before and after adsorption (Cu_1_Ni_2_HCF-Co^2+^).

Fig. 8a compares the results of Co^2+^ adsorption in electrochemical and conventional systems. The removal efficiency of electrochemical adsorption is 3–4 times higher than that of conventional adsorption, verifying that an electrochemical removal process had played an important role. And, the process of Co^2+^ adsorption involved both surface adsorption and cation intercalation/deintercalation.

The electrochemical adsorption capacity of CuHCF films is much higher than that of NiHCF films, verifying the results of integrated charge and EIS. The high adsorption capacity can be interpreted by the large lattice size of CuHCF. This is consistent with the fact that the sorption capacity of CuHCF is larger than that of NiHCF, as mentioned by Thierry *et al.*^54^ It is remarkable that the equilibrium removal efficiency of Cu_1_Ni_2_HCF films is higher than that of NiHCF films, and slightly lower than that of CuHCF films, which may due to the well-ordered porous structures of CuHCF films. The redox peaks of CuHCF film in Co^2+^ solutions are sharper than those of Cu_1_Ni_2_HCF film, indicating a faster dynamic process. The cycling stability of Cu_1_Ni_2_HCF film is obviously better than that of CuHCF film, when the equilibrium removal efficiency of Cu_1_Ni_2_HCF film is close to that of CuHCF film. That is why we chose Cu_1_Ni_2_HCF for the next step.

The crystal structure and surface morphology of Cu_1_Ni_2_HCF film before and after removing Co^2+^ were investigated by XRD and SEM; the results are depicted in Fig. 8b and c. Compared with the peaks of (In_1.88_Sn_0.12_)O_3_ (PDF # 89-4598) coated on substrates, the new peaks generated at around 17.95° and 25.35° and the sharper peaks at 35.48° and 37.65° are caused by Cu_1_Ni_2_HCF film. There is no significant change in the results of SEM and XRD after Co^2+^-loading, indicating that the structure of the nanocrystals had not been changed by the adsorption process. The concentrations of Cu^2+^ and Ni^2+^ before and after pretreating the films were determined by ICP-MS (Fig. S2†). The results show that there is no significant release of Cu^2+^ or Ni^2+^ from the films, indicating that the prepared nanoparticle films were stable as well. These phenomena are consistent with the expected cation insertion process, during which the essential feature of crystal structure is preserved. This may also due to the dilute solution and trace amount of Co^2+^ adsorbed on Cu_1_Ni_2_HCF. The same conclusions can be drawn from NiHCF and CuHCF films (as shown in Fig. S1†).

#### 3.2.3 Isotherm and kinetic studies

**Fig. 9:**
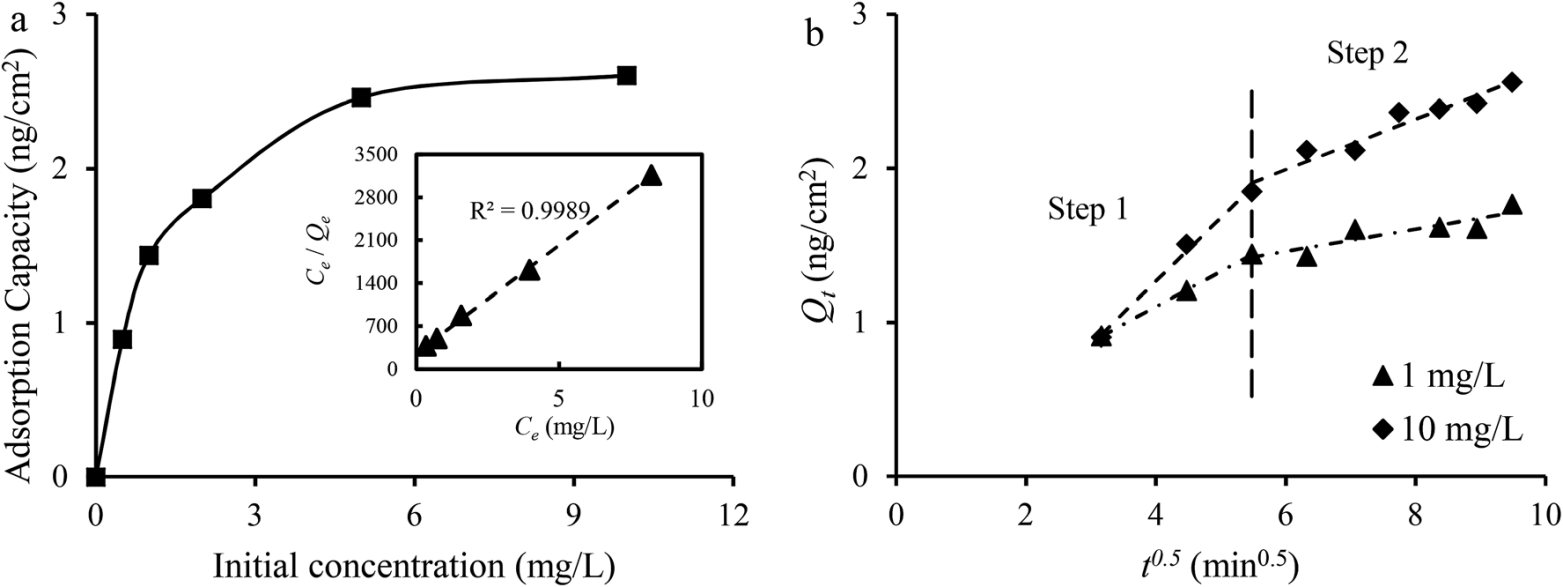
(a) The effect of initial concentration on the adsorption capacity. The inset is the fitted result with the Langmuir isotherm model; (b) adsorption kinetic data in 1 mg L^−1^ and 10 1 mg L^−1^ Co^2+^ solutions fitted with the intraparticle diffusion model.

The effect of initial concentrations on the adsorption capacity of Cu_1_Ni_2_HCF films for Co^2+^ was studied and the results are shown in Fig. 9a. It has been found that the plot reflected a two-stage process, a steep ascent portion followed by a plateau extending to the equilibrium. The main reason is that the concentration gradient between the solution and the interface of the adsorbent increased with a rise in initial concentration, which enhanced the driving force for the diffusion of Co^2+^ from solution to the films.^55,56^

The Langmuir isotherm model and the Freundlich isotherm model were used to fit the experimental data. The Langmuir isotherm model assumes that only one monolayer of adsorbate molecule can be adsorbed on homogeneous sites of the adsorbent and the adsorbates do not react with each other. The Freundlich isotherm model is based on multilayer adsorption on a heterogeneous surface. The calculation results of the *Q*_e_, constants and correlation coefficient (*R*^2^) are listed in Table 2. The linear correlation coefficient (*R*^2^) from the Langmuir isotherm model is 0.9989, which is higher than that calculated from the Freundlich isotherm model, and the value of *Q*_m_ from the Langmuir isotherm model is close to the practical *Q*_e_ obtained from experiment. It can be concluded that the Langmuir isotherm model can describe the adsorption process better than the Freundlich isotherm model, which is in agreement with the redox reaction. The results from the data in 1 mg L^−1^ Co^2+^ solutions fitted to the intraparticle diffusion, pseudo-first-order and pseudo-second-order kinetic models are given in Table 2. The sorption process involves bulk diffusion, film diffusion and intraparticle diffusion (or pore diffusion).^35^Fig. 8b shows that two linear regions exist in the intraparticle diffusion plot. The adsorption system was stirred at a speed of 500 rpm. Thus, the two curves may indicate that the adsorption of Co^2+^ onto Cu_1_Ni_2_HCF film occurred *via* two phases:^57^ film diffusion in the first linear part followed by intraparticle diffusion during the second linear portion. The linear portions of the two stages do not pass through the origin, and, *k*_p,1_ is much greater than *k*_p,2_, which suggests that both film and intraparticle diffusion processes are the probable rate-controlling steps.^58^ The constant *C*_1_ increased with an increase in ion concentration, indicating the growth of the thickness of the boundary layer. The probability of external mass transfer was reduced, and the probability of internal mass transfer increased conversely.^59^

**Table 2:** The parameters and correlation coefficients of isotherm models and kinetic models

Isotherm models	Parameters		Kinetic models	Constants	Initial concentration (mg L^−1^)	
					1	10
Langmuir	*Q*_m_ (mg cm^−2^)	0.0029	Pseudo-first-order	*Q*_e_ (mg cm^−2^)	0.0017	0.0062
	*K*_L_	1.3263		*k*_1_	0.0659	0.0044
	*R* ^2^	0.9989		*R* ^2^	0.9705	0.9083
Freundlich	*n*	3.0386	Pseudo-second-order	*Q*_e_ (mg cm^−2^)	0.0019	0.0079
	*K*_F_	0.0015		*k*_2_	45.0086	4.8711
	*R* ^2^	0.9370		*R* ^2^	0.9762	0.9730
			Intraparticle diffusion	*k*_p,1_	0.0002	0.0004
				*C*_1_	0.0002	−0.0004
				*R* ^2^	0.9998	0.9932
				*k*_p,2_	7 × 10^−5^	0.0002
				*C*_2_	0.0010	0.0010
				*R* ^2^	0.8084	0.9398

Both the pseudo-first-order kinetic model and the pseudo-second-order kinetic model show a good fit to the experimental data with well-matching correlation coefficients at a low initial concentration. At a higher initial concentration, the correlation coefficient (*R*^2^) obtained from the pseudo-second-order kinetic model is higher than that of the pseudo-first-order kinetic model, suggesting that the pseudo-second-order model is more suitable for describing the adsorption process. This may predict that electrochemical adsorption involved a complicated process, and chemical adsorption might be the rate-limiting step.^60^

## 4 Conclusions

In summary, the proportion of metals in Cu_*x*_Ni_*y*_HCF nanoparticle films can be controlled by the reactant ratio during the preparation process. With an increase in the Cu ratio, the crystallite size and *E*_f_ of Cu_*x*_Ni_*y*_HCF films varied from NiHCF films to CuHCF films. The results of SEM and XRD, together with the shape of the CV curves supported Cu^2+^ being inserted into the NiHCF lattice as countercations to maintain the electrical neutrality of the structure when the proportion of copper is less than 33%; at higher molar fractions of Cu, Ni was partially replaced by Cu. Cu_1_Ni_2_HCF showed superior electrochemical properties of stability and current responses. The electrochemical adsorption behavior of Co^2+^ followed the Langmuir isotherm model and the pseudo-second order model. Our study reveals the potential of Cu_*x*_Ni_*y*_HCF nanoparticle films as a promising technology for the recovery of Co^2+^ from spent LIBs.

## Conflicts of interest

There are no conflicts to declare.

## Supplementary Material

RA-009-C9RA00596J-s001
