# Supplementary material for: Preparation, characterization and application in cobalt ion adsorption using nanoparticle films of hybrid copper–nickel hexacyanoferrate
Source: RSC Adv. 2019 Mar 6;9(13):7485–94. doi: 10.1039/c9ra00596j (PMC9061196; doi:10.1039/c9ra00596j)
Supplement: RA-009-C9RA00596J-s001 [file RA-009-C9RA00596J-s001.pdf]

Electronic Supplementary Material (ESI) for RSC Advances.
This journal is © The Royal Society of Chemistry 2019

Journal Name

ARTICLE

Table S1 Slope values from the logarithmic plots of peak current vs. scan rate for Cu_xNi_yHCFs modified electrodes.

	CuHCF	Cu_4Ni_1HCF	Cu_2Ni_1HCF	Cu_1Ni_1HCF	Cu_1Ni_2HCF	Cu_1Ni_4HCF	NiHCF
Slope	0.7869	0.8776	0.8681	0.8634	0.7767	0.7512	0.7932

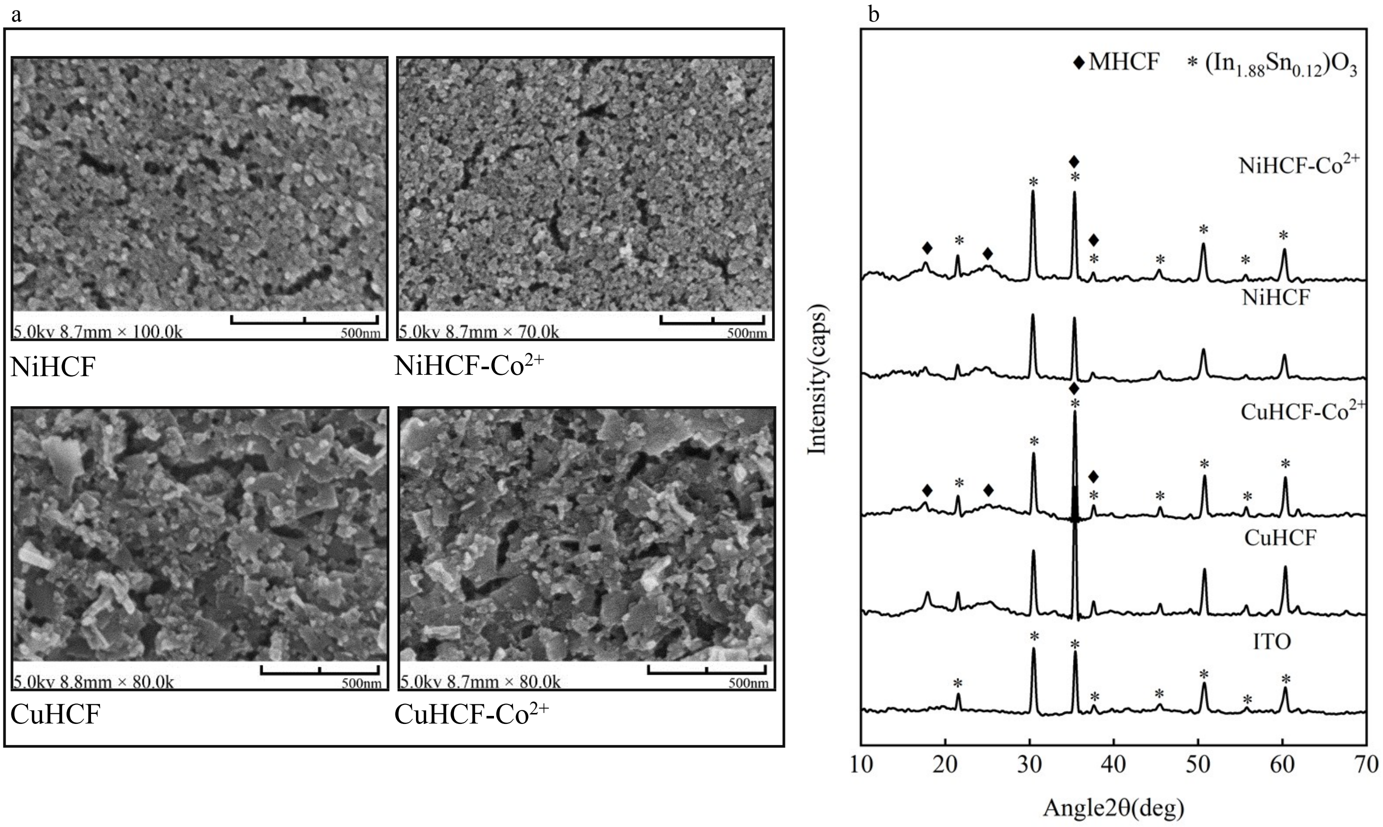

Figure S1 - (a) SEM images and (b) XRD patterns of MHCF (M: Cu, Ni) film before and after adsorption (MHCF-Co^{2+}).

Please do not adjust margins

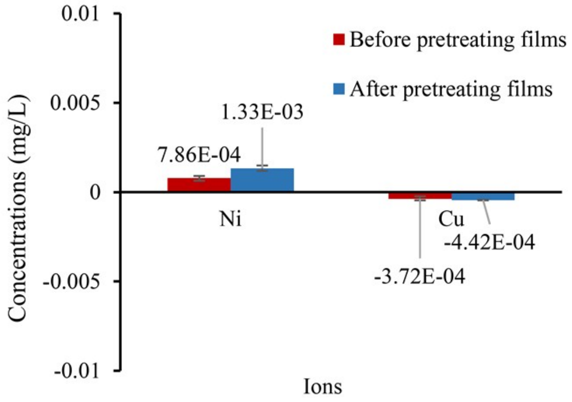

Figure S2 - Concentrations of K^+, Ni^{2+} and Cu^{2+} before and after pretreating Cu_1Ni_2HCF films in 1 mg/L Co^{2+} solutions.

This journal is © The Royal Society of Chemistry 20xx

Please do not adjust margins
